# Immunotherapy of food allergy: what is effective?

**DOI:** 10.1186/2045-7022-1-S1-S3

**Published:** 2011-08-12

**Authors:** Harald Renz

**Affiliations:** 1 Institute of Laboratory Medicine and Pathobiochemistry, Molecular Diagnostics, Marburg, Germany

## 

Allergic asthma as a complex outcome of pathogenic immunological, cellular and functional modifications of the airways is initiated by allergic reactions as a response mostly to inhalant allergens. Dysregulation of innate and adaptive immune functions contribute to the pathogenesis of the disease. A hallmark is the development of a Th2-driven inflammatory response in the airways. The underlying Th2-skewed balance is the result of a multi-functional process in which genetic predisposition and environmental exposures interact as major players. Maturation of the immune system already starts in utero, the most critical phase in the ontogenetic programming of the offspring. Endogenous as well as exogenous exposures may influence the maturation and differentiation of immune cells of the fetus and may thereby contribute to disorders such as allergies and asthma later on in life. Epigenetic mechanisms are proposed to mediate these effects. A comprehensive overview on the interaction of fetal exposures and the developing immune system will be provided that may contribute to or protect the progeny against the development of asthma. The new and exciting field of epigenetics will be highlighted with respect to T-cell differentiation and early allergic disease development. Furthermore, we emphasize new investigations that aimed to analyze fetal host innate immune responses to environmental microbial microorganisms and their possible future application in asthma protection.

